# Supplementary material for: How do associations between sleep duration and metabolic health differ with age in the UK general population?
Source: PLoS One. 2020 Nov 23;15(11):e0242852. doi: 10.1371/journal.pone.0242852 (PMC7682906; doi:10.1371/journal.pone.0242852)
Supplement: Attachment — Submitted filename: Response to Reviewers .docx [file pone.0242852.s002.docx]

Response to Reviewers

| **REVIEWER 1** | |
| --- | --- |
| REVIEWER COMMENT | AUTHORS’ RESPONSE |
| • Are there not more comprehensive citations for the relationship between sleep and metabolic risk factors from systematic reviews and meta-analyses? | Systematic review and meta-analysis citation has been added.  **Lines 18-19** |
| • What is meant by "increased dietary intake" (p.4, lines 17-18)? | ‘Dietary intake’ replaced with ‘dietary energy intake’ to make this more clear.  **Line 18** |
| • Although it is true that there have been few studies examining relationships between sleep and metabolic risk factors in younger samples, the authors fail to cite any relevant literature (e.g., Matthews et al, 2012; Javaheri et al, 2011). | Thank you for pointing this out – citations have been added.  **Lines 21-23** |
| • Please include citations and current guidelines re: sleep duration requirements across age groups (p.4, line 20). | Citations have been added for sleep duration requirements and change in metabolic health with age.  **Lines 24-26** |
| • p.5, line 26 - there are other dimensions of sleep beyond duration and quality, I would not make this statement so categorical. | Changed to reflect that these are two main metrics but not the only metrics.  **Line 31** |
| • The statements in p.5, lines 32-36 appear somewhat contradictory. | It was intended that one sentence refers to metabolic risk as an outcome and the following sentence refers to diet quality and eating behaviours. These are separate outcomes but the latter is likely to lie on the causal pathway between sleep and metabolic risk. Another sentence has been added to make this clear.  **Lines 42-43** |
| • I am somewhat confused by the statement "In order to develop appropriate recommendations among children and young adults" (p.5, lines 37-39) – your study did not include children below the age of 11, and the authors ultimately did not provide recommendations based on their results. | The reference to children has been removed. This was superfluous and as the reviewer correctly points out, this study was unable to draw conclusions on people aged below 11.  **Line 44** |
| • p.5, lines 43-45 – citations needed for the statement that "device-measured sleep is more precise"? Furthermore, are you not downplaying the importance of your own study? | Citation has been added. It could be argued that we are downplaying the importance but the intention of the sentence is to highlight and justify our choice of exposure variable. We accept that self-reported sleep duration is not as precise as device-measured sleep duration but the latter is currently infeasible for population-based studies like NDNS. We refer back to this in our discussion and conclusion, highlighting device-measured sleep duration as an avenue of future exploration.  **Line 52** |
| • The authors note that the present study focuses on sleep duration because it is "modifiable and relatively easy to record." That statement needs a citation. | Citation has been added.  **Line 49** |
| • Citation needed on p.5, lines 50-51 - metabolic syndrome/metabolic risk score is related to risk of what future health outcomes? | An example of a health outcome (cardiovascular disease) has been provided and a citation provided.  **Lines 58-59** |
| • Participants taking blood-pressure or lipid-lowering medications were excluded for analysis, which is good. Was data available on the % of participants who were taking medications for sleep purposes? | Unfortunately this data was not available. This has been added as a limitation  **Lines 283-286** |
| • Why were participants less than age 11 excluded (p.7, line 75), when the authors state that there is a need to "develop appropriate recommendations among children and young adults" (p.5, lines 37-39)? | A line has been added in the methods (Survey Design and Participants) to reflect the fact that metabolic data was unfortunately unavailable for participants aged<11 years.  **Lines 86-88** |
| • Would be helpful to know the % participants who were excluded due to age (<11 or >70 years), blood pressure or lipid-lowering medication use. | Further details have been added to ‘Characteristics of Study Population’ in results to provide the data about exclusion due to age. A line has been added in the methods (Survey Design and Participants) to reflect the lack of metabolic data for the youngest participants.  **Lines 86-88 and Line 188** |
| • What % of the sample were currently employed as shift workers? | Unfortunately this data is unavailable in the NDNS dataset. |
| • Table 1 – please provide raw data for individual metabolic risk factors, weekday sleep duration, and weekend sleep duration. | These have been added to Table 1.  **Line 198** |
| • The authors note that "the association appears strongest in the 36-50 age group" (p.12, line 203) – I would think that is better stated as the association was present only in the 36-50 age group, as no association exists in the other groups. | This change has been made.  **Line 216** |
| • Tables 2 and 3 – consider using a note to indicate which covariates were included in Models 1-3. | Notes are present in row two of the tables where it describes the model that yielded the results in each column.  **Line 221** |
| • The Results text and Table 2 uses phrases including "quadratic term", "squared term," and coefficient of age-adjusted sleep duration term2" – be consistent. | This has been changed to be consistent.  **e.g. Line 222 (and more)** |
| • p.13, line 213 – consider using the phrase "all ages" vs. "aggregated results" to be consistent with Table 2. | This change has been made.  **Lines 226-227** |
| • The authors provide the p value on p.15, line 241 – would be helpful if they provided p values for other significant findings noted in the Results text. | p values have been added for these results as requested by Reviewer 1. p values can also be obtained from the confidence intervals (<https://www.bmj.com/content/343/bmj.d2304>).  **Lines 215, 226, 239, 240, 255** |
| • The fact that the sample was largely white should be added as a limitation. | This has been added to the limitations. The fact that the sample is largely white is a result of the fact that our study population is representative of the UK population, which is largely white.  **Lines 277-281** |
| • Not sure what the authors mean by the sentence beginning "the recommendations change frequently with age..." (p.17, lines 294-295). Also provide citations. | Citation has been added and the sentence has been altered to make it easier to understand the meaning that the intervals at which recommendations change are narrower in these age groups.  **Lines 314-316** |
| • The authors found that the mid-age group appeared to be driving results for the effect of short sleep on metabolic risk but did not find significant results for the difference between weekday and weekend sleep in this age group. This is in contrast to evidence from Wong et al., who found that midlife adults who have greater social jetlag (difference in actigraphy-measured sleep between work and non-work days) showed a worse cardiometabolic profile. | We thank the reviewer for pointing out these research findings for comparison. The social jetlag described in Wong et al’s paper is not quite the same as the difference between weekend and weekday sleep duration in our paper. Wong et al define social jetlag as the difference in the midpoint of time of sleep on workdays and “freedays” – that is a measure of the timing of sleep rather than duration. Wong et al do measure sleep debt which is comparable to our metric – we have included this citation and added discussion about their findings.  **Lines 360-362** |
| • Provide citations for the sentences beginning "Screening for poor sleep..." and "There are a range of causes..." (p.20, lines 354-358). | Citations have been added.  **Lines 377-381** |
| • Provide age range for "young adults" (p.21, line 376). | Age range has been added.  **Line 402** |
| • Need more explanation for the first sentence in the Concluding Remarks section – what, specifically, might be the clinical implications of heterogeneity between age groups? | This has been altered to highlight both implications for designing clinical guidelines as well as for future research.  **Line 394-398** |
| **REVIEWER 2** | |
| REVIEWER COMMENT | AUTHORS’ RESPONSE |
| I think this will be a good addition to existing literature. Generalizability of the data may be difficult in this case since it was predominantly a white population, none the less the study was done thoughtfully. Authors have highlighted most of the potential limitations which one may think have. The tables are well done. The technique and the stats are well done with no major limitations. I like the fact that it draws more spotlight on weekend and weekday sleep variation which is an area of future research. | Thank you for these positive comments. We have added the limitation of a mostly white population to the Discussion. This was an important point and it was also raised by Reviewer 1. |
